# Information Circulation Among Spanish-Speaking and Caribbean Communities Related to COVID-19: Social Media–Based Multidimensional Analysis

**DOI:** 10.2196/42669

**Published:** 2023-08-23

**Authors:** Xanat Vargas Meza, Han Woo Park

**Affiliations:** 1 Institute for the Advanced Study of Human Biology Kyoto University Kyoto Japan; 2 Research and Development Centre for Digital Nature University of Tsukuba Tsukuba Japan; 3 Department of Media & Communication YeungNam University Gyeongsan si Republic of Korea; 4 Graduate Department of Digital Convergence Business and East Asian Cultural Studies YeungNam University Gyeongsan si Republic of Korea; 5 Cyber Emotions Research Center YeungNam University Gyeongsan si Republic of Korea; 6 Big Local Big Pulse Lab YeungNam University Gyeongsan si Republic of Korea

**Keywords:** COVID-19, social media, Spanish, multidimensional analysis, Caribbean, accessibility

## Abstract

**Background:**

Scientiﬁc studies from North America and Europe tend to predominate the internet and beneﬁt English-speaking users. Meanwhile, the COVID-19 death rate was high at the onset of the pandemic in Spanish-speaking countries, and information about nearby Caribbean countries was rarely highlighted. Given the rise in social media use in these regions, the web-based dissemination of scientific information related to COVID-19 must be thoroughly examined.

**Objective:**

This study aimed to provide a multidimensional analysis of peer-reviewed information circulation related to COVID-19 in Spanish-speaking and Caribbean regions.

**Methods:**

COVID-19–related, peer-reviewed resources shared by web-based accounts located in Spanish-speaking and Caribbean regions were identified through the Altmetric website, and their information was collected. A multidimensional model was used to examine these resources, considering time, individuality, place, activity, and relations. Time was operationalized as the 6 dates of data collection, individuality as the knowledge area and accessibility level, place as the publication venue and affiliation countries, activity as the Altmetric score and number of mentions in the selected regions, and relations as coauthorship between countries and types of social media users who disseminated COVID-19–related information.

**Results:**

The highest information circulation peaks in Spanish-speaking countries were from April 2020 to August 2020 and from December 2020 to April 2021, whereas the highest peaks in Caribbean regions were from December 2019 to April 2020. Regarding Spanish-speaking regions, at the onset of the pandemic, scientific expertise was concentrated on a few peer-reviewed sources written in English. The top scientiﬁc journals mentioned were from English-speaking, westernized regions, whereas the top scientiﬁc authorships were from China. The most mentioned scientific resources were about breakthrough findings in the medical and health sciences area, written in highly technical language. The top relationships were self-loops in China, whereas international collaborations were between China and the United States. Argentina had high closeness and betweenness, and Spain had high closeness. On the basis of social media data, a combination of media outlets; educational institutions; and expert associations, particularly from Panama, influenced the diffusion of peer-reviewed information.

**Conclusions:**

We determined the diffusion patterns of peer-reviewed resources in Spanish-speaking countries and Caribbean territories. This study aimed to advance the management and analysis of web-based public data from non-white people to improve public health communication in their regions.

## Introduction

### Colonized Lands and Colonized Internetscapes

Aspects, such as climate change, precarious health care infrastructure, undermining of scientiﬁc expertise, slow or negligent government response, contradictory or misleading information, and opposing societal values, have worsened the impact of COVID-19 worldwide [1-5]. For example, COVID-19 deaths per 100,000 inhabitants in Peru (102.89), Bolivia (72.16), Chile (69.89), Spain (69.69), Ecuador (68.74), and Mexico (65.56) were among the top 10 worldwide [6]. Even after the vaccination schemes began, Peru (529.69), Colombia (215.17), Argentina (212.72), and Paraguay (187.87) were among the top 20 countries [7]. All of them are Spanish-speaking countries, mostly located in Latin America.

American-led and European-led studies published in English tend to dominate the web-based landscape, partly because English is the most used language on the internet [8]. This implies that web-based health communication is beneﬁcial and fast for English-speaking communities. Nonetheless, it appears that Caribbean countries that are geographically close to Latin America initially had effective COVID-19 management [9], with deaths per 100,000 inhabitants in each country ranging from 1.08 to 26.45 [6]. To better understand such disparities, how academic inquiries related to public health are curated and disseminated by Spanish-speaking and Caribbean communities on the internet must be rigorously investigated. Through this, we can better clarify the impact of social media in terms of communication during global health crises.

The rest of this paper is organized as follows. We have provided a cultural context for Spanish-speaking and Caribbean regions, reviewed relevant social media–related literature, identified research gaps, and defined our objectives. Then, we have described our methods and results, followed by a discussion and a summary of the conclusions.

### Culture and Landscapes

Culture can be deﬁned as shared or communal knowledge and practices [10]. There exist complexities unique to the communities discussed in our paper, as many formerly colonized territories continue to fight for recognition and autonomy. Displacement, forced labor, rape, and slavery led to “mestizaje,” a mixture of races. Consequently, cultural and territorial markers are often confused, misplaced, or omitted when identifying such communities.

The term, “Hispanic,” refers only to cultures related to Spain, the original location of the Spanish language (also called Castillian). This group comprises 20 countries and territories situated in North, Central, and South America; the Caribbean; and Spain. These communities, excluding Spain, makeup Latin American communities. Latino and Latina territories include the same 19 territories plus 7 others—Brazil, French Guiana, Guadeloupe, Martinique, Haiti, Saint Barthelemy, and Saint Martin—located in the American continent and the Caribbean, with a high prevalence of African and other immigrant roots among their populations. These countries speak several other languages, with adaptations and fusions with local languages.

Spanish is the second most spoken language in the world, with approximately 493 million native speakers [11]. However, classiﬁcations deeply rooted in national boundaries ignore the wide diaspora of Spanish-speaking communities worldwide. Hence, our study explored communities beyond national limitations while recognizing that geographical location might also play a relevant role in health communication.

### Informationscapes

The COVID-19 pandemic has drawn various reactions worldwide. A crowdsourced ﬁlm [12] documented the outbreak in Wuhan, China, the epicenter of the pandemic. Moreover, ordinary citizens who realized that they had or might have had COVID-19 created web-based groups and distributed surveys to identify short-term and long-term symptoms before experts mobilized in these spaces.

In recent years, extensive studies about communication through social media have focused on the United States. Consumers of fake news are more active in the publication of content than evidence-based consumers [13]. At a given time, an American citizen was the largest source of COVID-19 misinformation in English [14], and 12 individuals and their organizations were identiﬁed as hubs against vaccines on Facebook and Twitter [15]. Among them, 2 were non-white people, but none of these individuals were from our territories of interest.

There is evidence suggesting that people actively used social networking sites to ﬁll information gaps during the onset of the pandemic [16]. Pascual-Ferrá et al [17] measured COVID-19 networks on Twitter and found that the World Health Organization, an expert, and an American citizen spreading fake news were the most central hubs at the onset of the pandemic. Ahmed et al [18] analyzed the “ﬁlmyourhospital” hashtag on Twitter, where the most important hubs were ordinary citizens, and YouTube was the most linked information source. They also probed the “5Gcoronavirus” hashtag on UK Twitter [19], where disinformation was not fought by any leader, fake news websites were frequently visited as information sources, and few people believed in the conspiracy. Pulido et al [20] analyzed 1000 Chinese and English tweets about the COVID-19 outbreak for 2 days and discovered that false information was tweeted more but retweeted less than science-based evidence or fact-checking tweets, whereas science-based evidence and fact-checking tweets captured more engagement than mere facts. Furthermore, Park et al [21] explored news framing, and Park et al [22] documented Twitter use related to COVID-19 in South Korea.

Among scientiﬁc inquiries related to our regions of interest, Islam et al [23] assessed agency websites, Facebook, Twitter, and web-based newspapers between December 2019 and April 2020 in 25 languages from 87 countries and found that 82% of the texts included false information. Zhu and Park [24] proposed a quadruple helix model to assess COVID-19–related information circulation. However, these studies omitted several countries in Latin America, the Caribbean, and Africa.

Meanwhile, Massachusetts Institute of Technology [25] conducted a survey about COVID-19 beliefs, behaviors, and norms. The survey revealed that, first, >50% of respondents from Chile, Panama, and Puerto Rico trusted government communications; Nicaraguans trusted World Health Organization personnel; and Brazilians trusted journalists. Second, no clear trusted authorities were found for the rest of the surveyed countries of interest. Third, a general low exposure to trustful information was observed. A study about the perception of fear and exaggeration fueled by Peruvian media described social media and television as the most frequent sources [26]. Furthermore, global studies of academic responses to COVID-19, including a few of our target countries, found that there was insufficient expert consultation and equal concern for climate change by governments, whereas Argentina, Brazil, and Chile reported the highest disruption to their work [27].

A common research approach for the dissemination of COVID-19 fake news is infodemiology [28], which implies that information is a living organism that infects an unwilling host. Moreover, truth-based narratives that provide emotional benefits and are aligned with the individual’s core values can motivate people to behave productively [29]. Some social media platforms, such as Twitter and Facebook, deployed warnings about false narratives, which is a technique called “prebunking” and roughly analogous to vaccination [30]. Similarly, Schillinger et al [31] proposed a model to assess the effects of social media, such as contagion, vector, surveillance, inoculant, disease control, and treatment, which considers information as an integral organism coexisting with human and digital communities.

### Gaps and Opportunities

The characteristics of fake news dissemination and its transmitters in the English language create the illusion that misinformation is more prevalent than it actually is among populations, whereas some indication exists that exposure to reliable information about COVID-19 may be insuﬃcient in Spanish-speaking and Caribbean countries. To execute communication strategies effectively, social network analysis can be used to detect relevant hubs that disseminate reliable information. Furthermore, most studies intersecting social media and prebunking are conducted in westernized, educated, industrialized, rich, and democratic (WEIRD) countries, whereas studies that incorporate data from some non-WEIRD communities can mischaracterize entire regions. This indicates a gap in the comprehension of how reliable information works among non-WEIRD communities. Therefore, reliable information dissemination paths must be identified through social media among non-WEIRD communities, which may provide fast access to key information during the COVID-19 pandemic.

### Study Objectives

Given the gaps and opportunities found in our literature review, we examined the dissemination of COVID-19–related reliable information on social media among Spanish-speaking communities and Caribbean communities.

## Methods

### Datascapes

#### Altmetric Data

Several analytical techniques target scientiﬁc communication. Scientometrics uses quantitative factors and models to investigate information deemed valuable by scientiﬁc authorities. To determine how scientiﬁc information (ie, reliable information) is disseminated to the general public, we focused on the Altmetric website [32]. This platform has sourced data from the web since 2012, including citations of scientiﬁc studies on Wikipedia, public policy documents, blogs, mainstream media coverage, bookmarks on reference managers, and social media mentions.

Altmetric data allow for the collection of timely records of the attention, dissemination, inﬂuence, and impact of research papers, which are more diverse than conventional citation measurements and can be applied beyond journal articles and books, as it includes scientiﬁc articles in reputed magazines. Given that literacy levels in our regions of interest vary signiﬁcantly, that their use of social media is increasing rapidly, and that Spanish-based studies are not strongly considered in conventional citation indexes, diverse data sources, such as altmetrics, might be able to capture the dissemination of recent scientiﬁc information accurately in our case study. Thus, we defined a search query as described in the following section.

#### Search Query

To prioritize scientiﬁc resources and because COVID-19 is a health-related issue, we requested the Altmetric system to search for social media mentions of resources available in PubMed. PubMed is a database created by the National Center for Biotechnology Information of the United States, which was inaugurated in 1996. It contains >34 million citations and abstracts about biomedical resources [33]. We used the following keywords: “coronavirus OR covid-19” and requested all outputs. Further ﬁlters included date and country and region, as described in the following section.

#### Classification and Selection of Countries and Regions

We combined several data sources to summarize the population, status of the Spanish language, number of Spanish speakers, and percentage of Spanish speakers per population in each country and region of the world when such information was available (Multimedia Appendix 1 [34-38]). Next, we identified the Spanish-speaking (labeled as S in the *category* column of Multimedia Appendix 1 [34-38]) countries and territories by acknowledging where the language is considered as a ﬁrst or second oﬃcial language. By looking at the small quantity of Spanish comments in altmetric social media mentions, we discarded the following countries with many Spanish speakers: Brazil, France, Germany, Italy, the Philippines, the United Kingdom, and the United States. Finally, Caribbean (labeled as C in the *category* column of Multimedia Appendix 1) countries were selected because they are geographically close to the equatorial line, including the oceans surrounding the American or Abya Yala continent. Once the countries and regions were identified, we proceeded with the analysis.

### Analysis Techniques

#### Multidimensional Model for Peer-Reviewed Resources in Social Mediascapes

**Figure 1:**
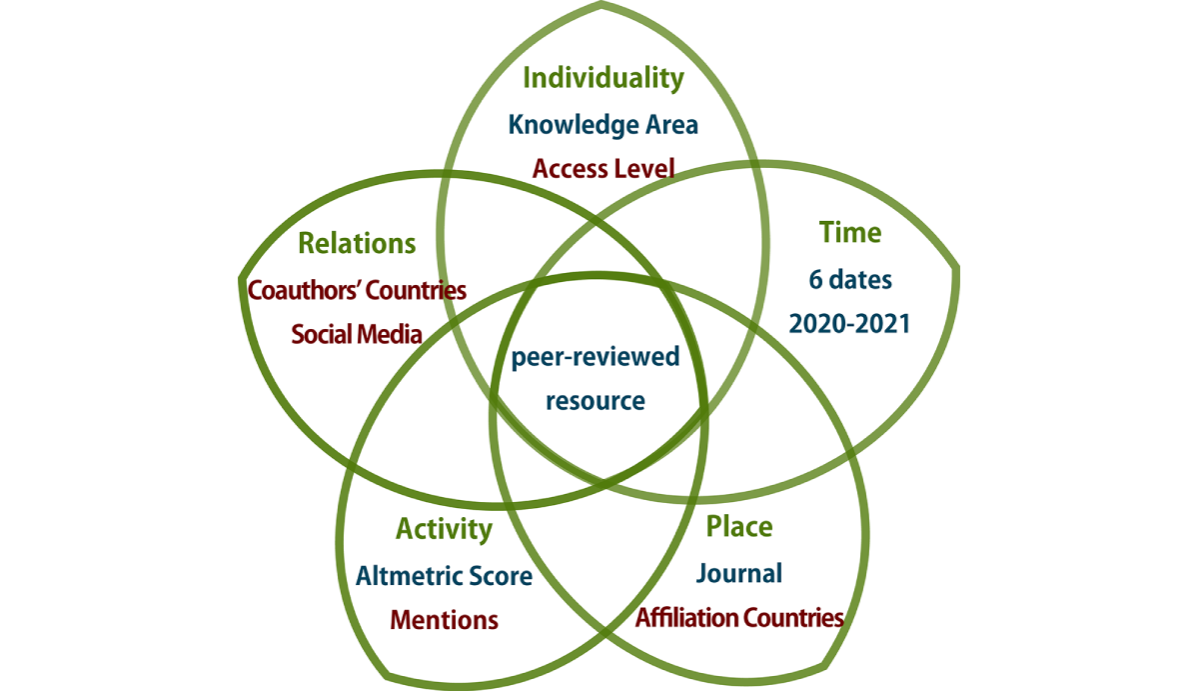
Model for peer-reviewed resources on the social mediascape.

Altmetric data provide initial useful indications for disseminating reliable information on social media. However, if such data are examined considering multiple factors, that is, if each publication is treated as a complex media object, the analysis can provide a more comprehensive overview of the communication patterns among related actors [39]. Figure 1 adapts the analysis model from the paper by Vargas Meza and Yamanaka [39]. Factors written in blue were obtained directly from the Altmetric website, whereas factors written in red were veriﬁed by an author.

The studied entity was each peer-reviewed resource extracted using altmetrics. The 5 main dimensions of the entities are included in Textbox 1 and are thoroughly defined in the subsequent sections.

**Description of the 5 dimensions of peer-reviewed resources.**

- The individuality dimension comprised knowledge area and accessibility.
- The time dimension is the data collection points.
- The place dimension was considered as the journal name and the country where each author’s aﬃliation is located.
- The activity dimension comprised the Altmetric score and social media mentions in the countries and regions of our interest.
- The relations dimension was considered on the basis of the aﬃliation countries of coauthors and on the social media users’ data (when available).

#### Considerations for the Individuality Dimension

The knowledge area was extracted from the subject column obtained from the Altmetric data, whereas accessibility refers to the availability and access level of the peer-reviewed resource. This was based on 4 aspects of the peer-reviewed resources that were veriﬁed through a regular internet connection: golden access (available from the journal), green access (available from a document repository), Google access (the resource appears on the ﬁrst page of Google Search results), and availability of a translated abstract in any language.

#### Considerations for the Time Dimension

Although Chinese authorities disclosed information regarding COVID-19 through oﬃcial channels on December 31, 2019 [40], some researchers have suggested that the virus was documented in early December 2019 [41]. Therefore, Altmetric data were collected 6 times, adjusting the mention period across 4 months: December 1, 2019, to April 1, 2020; April 2, 2020, to August 2, 2020; August 3, 2020, to December 3, 2020; December 4, 2020, to April 4, 2021; April 5, 2021, to August 5, 2021; and August 6, 2021, to December 6, 2021.

#### Considerations for the Place Dimension

The name of the journal was extracted from the Altmetric data column called “Journal or Collection Title.” Some coauthors’ aﬃliation countries were included in the data column called “Aﬃliations.” However, because such aﬃliations often did not include institutions, such as hospitals, military medical centers, companies, or news agencies, each data entry was manually checked by visiting the peer-reviewed resource and creating a new data column titled “Aﬃliation Country.”

#### Considerations for the Activity Dimension

The Altmetric score is a global estimation of the social media attention given to peer-reviewed resources. To obtain more speciﬁc data, we also counted social media mentions of the resource in our selected regions, extracted from the column “Number of Mentions” in each country’s data set.

#### Consideration for the Relations Dimension

To understand international research collaboration related to COVID-19, a relations network was constructed based on the country or region where each peer-reviewed source authors’ aﬃliation was located. Meanwhile, Altmetric data contain some social media details, including the outlet or author that shared the source if they managed a public account. By checking the original account and its oﬃcial web page (if any), we classiﬁed the social media accounts according to the scheme in Table 1.

**Table 1:** Categories and subcategories of social media accounts.

Category	Description	Subcategories
Association	Professional associations	Hospital association, international association, medical association, and scientiﬁc association
Community	Civilian communities	Community Facebook page
Education	Educational outlets	Facebook page, hospital, national library, research institution, university, and university Facebook page
Enterprise	For-profit firms	Directory, laboratory, and pharmacy
Hospital	Health service providers	Clinic and institute
Individual	Common citizens	Journalist and physician
Media	Media companies	Blog, magazine, migrated media, new media, news agency, newspaper, portal, radio station, TV channel, and TV news channel
NGO^a^	Nonprofit organization	Organizations not for profit
Unknown	Uncleared items	All other items

^a^NGO: nongovernmental organization.

#### Social Network Analysis

Social network analysis is a set of methods and techniques used to understand social relations and their structures [42]. We used this technique on the relations dimension of the Altmetric data to provide an overview of scientiﬁc, international cooperation reflected in the COVID-19 information circulating in our regions of interest. Therefore, countries and regions that cooperated in each peer-reviewed resource were quantiﬁed, their basic degree centralities were calculated, and their relations were drawn using Gephi software (version 0.7.0) [43].

### Ethical Considerations

This study was considered exempt from ethical review because it was conducted on scientific studies records publicly available on the internet. As such, it did not interfere with human data beyond measuring internet activity.

## Results

### Time Dimension—Mentions Across Time

**Figure 2:**
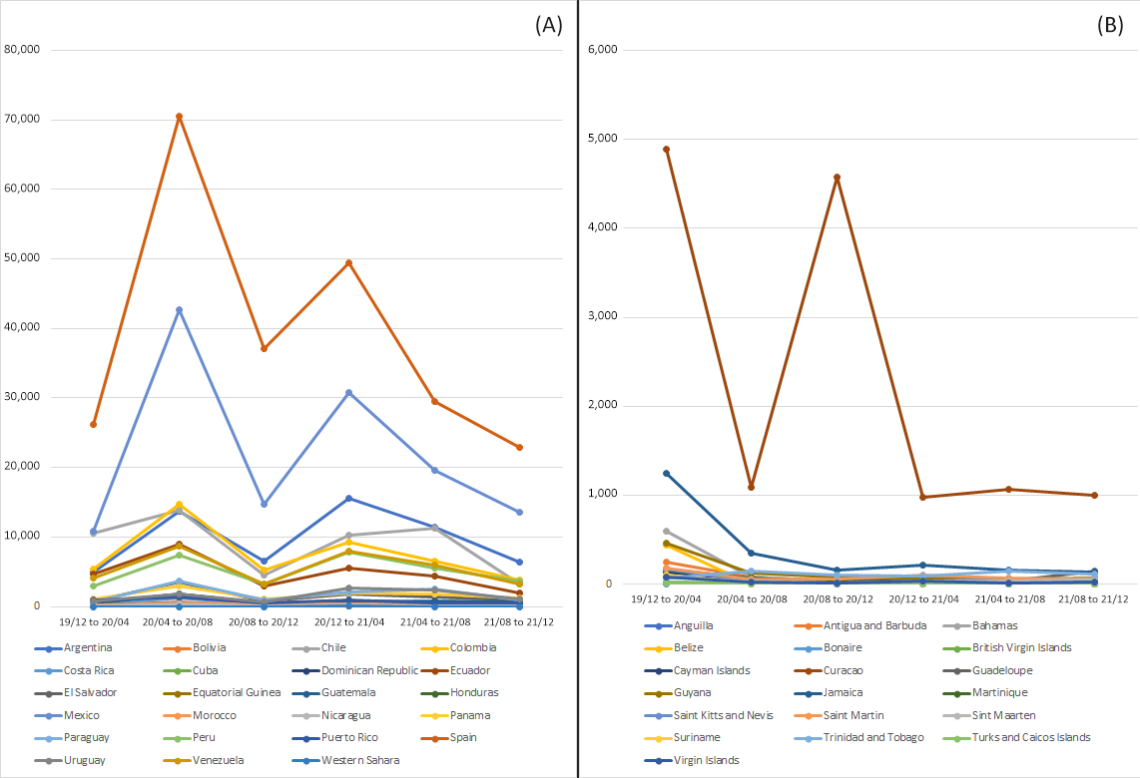
Mentions in (A) Spanish-speaking and (B) Caribbean countries across time.

Peer-reviewed resources were mentioned 685,560 times in Spanish-speaking countries and regions. Figure 2A shows that these resources tend to follow the same trends, with 2 high peaks and a constant decline after April 2021. The highest peaks occurred from April 2020 to August 2020 and from December 2020 to April 2021. The ﬁrst attention peak corresponds to the ﬁrst COVID-19 wave across Latin America that was initiated with particular infectiousness in Bolivia, Chile, Ecuador, Panama, and Peru; however, such trends do not correspond to infection patterns in Spain and Mexico or the world average [44]. Furthermore, countries with consistently high number of mentions were Spain and Mexico. This implies that social media agents in the 2 countries responded rapidly to the information needs in other regions that share a common language.

Regarding the second information peak, it corresponds to infectious waves in Argentina, Bolivia, Chile, Colombia, Costa Rica, Mexico, Panama, Paraguay, Peru, Spain, and Uruguay; however, the Mexican wave was not as prominent as in the rest of the mentioned countries [44]. Figure 2A shows that Argentina was one of the countries with the highest mentioned peaks between December 2020 and April 2021 (third place), corresponding to one of their infection waves.

A total of 21,912 mentions of peer-reviewed resources were identified in Caribbean countries and regions. Figure 2B shows that only 1 relevant peak occurred from December 2019 to April 2020. This communication pattern does not correspond to infection patterns across these countries and territories or with the world average [44], as infection rates have been increasing across time, although with disparities. Moreover, Curacao is the only country that does not ﬁt the general communication pattern and has the highest number of mentions; it has 2 communication peaks, the second peak coinciding with the ﬁrst relevant infection wave in Curacao.

### Spanish-Speaking Countries’ Resources Across Time (December 2019 to April 2020)

From this section onward, we have focused on the data collected throughout the ﬁrst period in Spanish-speaking countries. A total of 76,349 mentions were found. Most peer-reviewed sources in this period were published in 2020 and March (Multimedia Appendix 2). A few sources dating back to the 1970s, 1980s, and 1990s and a couple hundred sources from the 2000s and 2010s were also circulating. In the next section, we examine the types of resources and their accessibility.

### Individuality Dimension of Peer-Reviewed Resources in Spanish-Speaking Countries

A total of 1783 peer-reviewed resources were mentioned on social media during the ﬁrst period of data collection. These 1783 resources can be divided into 1697 (95.18%) scientiﬁc papers, 68 (3.81%) news articles, 9 (0.5%) book chapters, 6 (0.34%) audio interviews, 2 (0.11%) books, and 1 (0.06%) infographic that received peer reviews from a scientiﬁc journal. Therefore, despite most resources being scientiﬁc papers, there were alternatives, such as short interviews with experts and an infographic, that were easy to understand for the general public.

The topics of the sources were organized into 74 different knowledge areas. The most numerous were in the medical and health sciences area, including a small subset (<1%) in the psychology and neuroscience area (Multimedia Appendix 3). However, although nursing is an integral part of public health ﬁrst responses, only 0.56% (10/1783) of peer-reviewed resources related to this ﬁeld were mentioned on social media.

Regarding accessibility, most peer-reviewed resources were written in English (1774/1783, 99.49%), followed by Spanish (6/1783, 0.34%), Portuguese (2/1783, 0.11%), and Norwegian (1/1783, 0.06%). Table 2 shows that most resources were available either directly from the journal that published them (golden access) or through a document repository (green access). However, very few abstracts were counted in other languages, and only 1.4% (25/1783) had the highest accessibility score. Most of these resources (10/25, 40%) were authored by researchers located in China; mostly published by the *New England Journal of Medicine*; and mentioned on Twitter, Facebook, and other news media platforms. This suggests that the authors who conducted breakthrough studies about COVID-19 considered ways to make their results accessible worldwide. Thus, publishing venues and affiliation locations are essential, as described in the following section.

**Table 2:** Accessibility of peer-reviewed resources.

	Golden access	Green access	Google access	Multilingual abstract
Resources (N=1783), n (%)	1671 (93.71)	1338 (75.04)	1764 (98.93)	57 (3.19)
Values, median (range)^a^	1 (0-1)	1 (0-1)	1 (0-1)	0 (0-1)
Values, mean (SD)^b^	0.9371 (0.2426)	0.7504 (0.4328)	0.9893 (0.1027)	0.0319 (0.1760)

^a^Total=3.

^b^Total=2.7089 (SD 0.5349).

### Place Dimension of Peer-Reviewed Resources in Spanish-Speaking Countries

Peer-reviewed resources from 586 scientiﬁc journals are mentioned. The top 10 scientific journals mentioned were from westernized backgrounds, such as the United States and the United Kingdom (Multimedia Appendix 4). Therefore, most of the scientiﬁc resources circulating in Spanish-speaking countries through social media were likely written in English.

Peer-reviewed resources were written by authors located at institutions in 85 countries and territories (Multimedia Appendix 4). The ﬁrst breakthrough country (China) was at the top in terms of authorships found in mentions at the onset of the COVID-19 pandemic, followed by English-speaking countries. In contrast, a few Spanish-speaking countries’ authorships were found in this period. Among them, authorship can be detected from Spain (56/4073, 1.37%); Colombia (26/4073, 0.63%); Mexico (15/4073, 0.36%); Venezuela (9/4073, 0.22%); Argentina (7/4073, 0.17%); Chile, Honduras, and Peru (6/4073, 0.14%); Ecuador (4/4073, 0.09%); Bolivia, Morocco, Panama, and Paraguay (2/4073, 0.04%); and Puerto Rico; and Uruguay (1/4073, 0.02%). Thus, a gap in the circulation of scientific information in Spanish-speaking contexts can be noted.

### Activity Dimension of Peer-Reviewed Resources in Spanish-Speaking Countries

Table 3 describes the top 10 mentioned scientiﬁc papers in Spanish-speaking countries and regions during the ﬁrst data collection period. All of them (10/10, 100%) were under the category of medical and health sciences. Of those 10 papers, 5 (50%) have authors with aﬃliations in China, 3 (30%) in Hong Kong, 3 (30%) in the United States, 1 (10%) in Germany, and 1 (10%) in the United Kingdom. Most of these papers (8/10, 80%) are about breakthrough ﬁndings, descriptions, and management of patients with COVID-19, with recent publication dates and involving a high level of specialized language. Only 1 resource is related to coronavirus in general and was published in 2007. A high number of mentions in Spanish-speaking countries did not always correspond to a high Altmetric score.

Most of the abovementioned peer-reviewed resources (1697/1783, 95.18%) in Spanish-speaking territories are scientiﬁc papers. However, resources also contained some news articles. Table 4 provides details about the top 11 news articles during our ﬁrst data collection period. The first 2 are on par with the top mentions of scientiﬁc papers. The second news article refers to the death of Ugandan–South African scientist Gita Ramjee owing to COVID-19. This top news article is the only one that is no longer available. The 11 resources versed on high-interest topics for the world at large, including advice for governments and medical workers and graphical information that facilitates accessibility for a large reading base if they understand English. They were released by *Nature* between February 2020 and April 2020, and only the ﬁfth article was counted with identiﬁed aﬃliations in Ireland. A discrepancy also exists between the number of mentions in Spanish-speaking regions and Altmetric scores. To better understand the reason for this discrepancy, we have described scientific collaboration and social media hubs in the next section.

**Table 3:** Top 10 scientific papers mentioned in Spanish-speaking countries and regions.

Mentions, n	Title	Publication date	Altmetric score
3109	Aerosol and surface stability of SARS-CoV-2 as compared with SARS-CoV-1	March 13, 2020	21,139
2318	COVID-19: protecting health-care workers	March 1, 2020	3286
1873	Persistence of coronaviruses on inanimate surfaces and their inactivation with biocidal agents	March 1, 2020	11,964
1845	Substantial undocumented infection facilitates the rapid dissemination of novel coronavirus (SARS-CoV2)	March 16, 2020	15,269
1541	Viral dynamics in mild and severe cases of COVID-19	March 1, 2020	4108
1470	Severe acute respiratory syndrome coronavirus as an agent of emerging and reemerging infection	October 12, 2007	8748
1389	Treatment of 5 critically ill patients with COVID-19 with convalescent plasma	March 27, 2020	13,761
1144	A trial of lopinavir–ritonavir in adults hospitalized with severe Covid-19	March 18, 2020	7613
930	Characteristics of and important lessons from the coronavirus disease 2019 (COVID-19) outbreak in China	February 24, 2020	11,253
878	A novel coronavirus from patients with pneumonia in China, 2019	January 24, 2020	4478

**Table 4:** Top 11 news articles mentioned in Spanish-speaking countries and regions.

Mentions, n	Title	Publication date	Altmetric score
4160	Coronavirus: three things all governments and their science advisers must do now	March 17, 2020	4573
1162	Coronavirus latest: scientists mourn renowned HIV researcher who died of COVID-19	April 6, 2020	7445
356	Mystery deepens over animal source of coronavirus	February 26, 2020	2619
342	Why does the coronavirus spread so easily between people?	March 6, 2020	2595
255	Fast, portable tests come online to curb coronavirus pandemic	March 23, 2020	1332
238	How blood from coronavirus survivors might save lives	March 24, 2020	2459
1389	What China’s coronavirus response can teach the rest of the world	March 17, 2020	1913
1144	Covert coronavirus infections could be seeding new outbreaks	March 20, 2020	3230
930	The coronavirus pandemic in ﬁve powerful charts	March 18, 2020	1229
878	Coronavirus lockdowns have changed the way earth moves	March 31, 2020	3464
178	How much is coronavirus spreading under the radar?	January 24, 2020	4478

### Relations Dimension of Peer-Reviewed Resources in Spanish-Speaking Countries

The top research authorship countries, in terms of degree centrality, are listed in Table 5. Chinese authorship was consistently high regarding coauthorship (degree) and fast access to authorship (closeness) and as a bridge between groups of countries (betweenness). Geographically close territories, such as South Korea, Singapore, and Hong Kong, were also relevant in terms of degree and betweenness. Furthermore, 2 Spanish-speaking countries were included in Table 5—Argentina with high closeness and betweenness and Spain with high closeness. The data revision revealed that Argentina had more authorship connections with Spanish-speaking regions than Spain, despite their low degree of 122. Spain was among the fastest respondents in terms of scientific authorship, whereas Argentina was among the most diverse and fastest respondents in terms of coauthorship with other Spanish-speaking countries. Owing to the mixture of top English- and non–English-speaking country affiliations, information circulation metrics likely varied in terms of the Altmetric score.

**Table 5:** Top-10 degree centralities of country affiliations.

Country	Degree	Country	Closeness	Country	Betweenness
China	8049	The United States	0.9303	The United States	0.1831
The United States	6199	The United Kingdom	0.8670	The United Kingdom	0.0865
The United Kingdom	2111	China	0.8354	China	0.0641
Italy	1769	Germany	0.8101	Italy	0.0508
Germany	1250	Italy	0.7974	Germany	0.0331
South Korea	1177	France	0.7848	France	0.0325
Canada	1162	Argentina	0.7700	Switzerland	0.0314
France	1100	The Netherlands	0.7658	Singapore	0.0248
Saudi Arabia	989	Switzerland	0.7637	Argentina	0.0236
Singapore	722	Spain	0.7405	Hong Kong	0.0208

**Figure 3:**
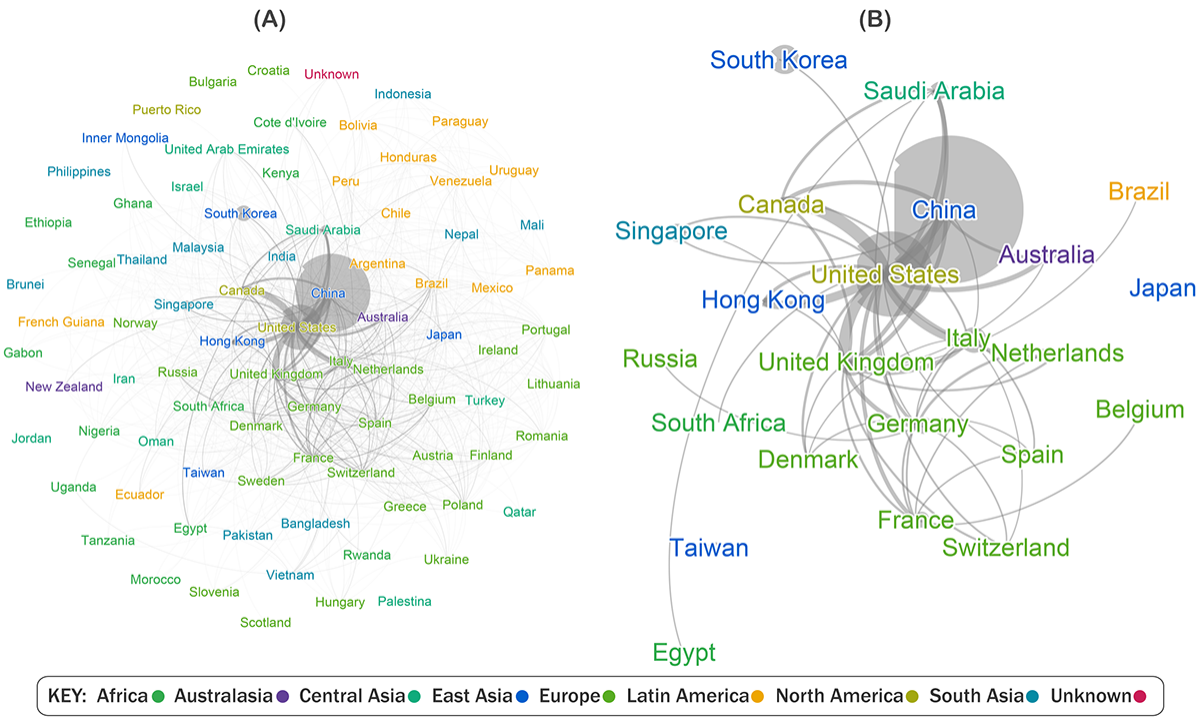
Affiliation countries cooperation—(A) scientific collaboration among countries and (B) top countries with scientific collaborations.

Figure 3 shows undirected unipartite networks drawn using the Fruchterman-Reingold algorithm. They displayed all the collaborations found in the peer-reviewed resources belonging to the first data collection period and the most frequent collaborations. Tie thickness corresponds to the frequency of such collaborations, including in-country collaborations (1-3407 and 92-3407 in Figure 3), and color corresponds to the geographical region. This classiﬁcation does not correspond to our previous country classiﬁcation scheme, as we emphasize the geographical location in the ﬁgure. Most scientific authorships were in the same country, suggesting that international cooperation was not prominent at the onset of the COVID-19 pandemic. Furthermore, the most frequent international collaborations were between China and the United States.

Regarding Spanish-speaking countries, it can be assumed that, apart from Spain, the rest of the Spanish-speaking territories were on the periphery of scientiﬁc contributions at the onset of the COVID-19 pandemic. The representation of Caribbean countries is even lower, whereas Brazil is among the core scientiﬁc contributors. Such results suggest the lack of a local context in COVID-19–related peer-reviewed resources circulating in the Spanish-speaking and Caribbean regions.

Some indications of Spanish-speaking sources were found through the data openly available on Twitter (Multimedia Appendix 5). Of the 143 sources, 55 (38.5%) were from Spain, 25 (17.5%) from Mexico, and 17 (11.9%) from Colombia. Most sources (79/143, 55.2%) were media outlets, whereas educational institutions, such as universities and expert associations (16/143, 11.2% each), were also relevant. We also noted that most of the top Twitter sources were from Spain. Traditional media, such as newspapers and radio that migrated on the web, and media that were born on the internet (new media) were found among them. Hospitals and expert associations were also highlighted. Furthermore, the identiﬁable source that tweeted the most about COVID-19 during our ﬁrst analysis period was a blog from Panama, emphasizing the role of this country’s media in the diffusion of health information in Spanish. Panama is an information hub connecting North, Central, and South Abya Yala, which allowed it to bridge COVID-19 information, in addition to having excellent access to information for managing a high number of infections.

## Discussion

### Scientiﬁc Communication and Information Access

Experts were among those involved in the early communication about COVID-19 on the web, including scientists stationed in the breakthrough country, who provided information in multiple languages through multilingual abstracts of their investigations. This is not a common practice that would be worthwhile for the sake of 2 types of public: scientiﬁc experts who are not familiar with the language or the knowledge ﬁeld of their peers and common people. Communities increase their internet access every year, with >65% of Spanish-speaking and Caribbean territories already connected [45]. Therefore, social media–based communication by scientiﬁc experts can increase the health beneﬁts for patients with COVID-19 in these regions.

The peer-reviewed content was not only available in text format but also in audio and image formats. The increasingly important role of scientiﬁc communication in the public and scientiﬁc ﬁelds can be noted here. With trends, such as immersive journalism, which uses dynamic web pages and audiovisual resources, scientiﬁc journals can adopt similar tools to highlight breakthroughs or relevant topics.

Another aspect was the fall of paywalls, as most COVID-19–related studies and news were available directly from their original publication sources. So far, COVID-19–related information remains accessible; however, whether this situation is permanent remains unclear. Although some indication exists that open access beneﬁts scientiﬁc citations [46], the increasing monopoly of news agencies and major news outlets may imply more commodiﬁcation of recent information. The COVID-19 pandemic falls into the public emergency category of reasons for avoiding paywalls; however, it may also lead to audience development and wide access [47]. In addition, there is evidence suggesting that between 10% and 87% of people infected with SARS-CoV-2 developed a chronic illness known as long COVID (also known as post–COVID-19 condition) [48]. Therefore, media outlets must consider the long-term informational needs of patients living with an emergent disease.

The difference between information accessibility and understanding should be addressed. Van Dijk [49] considered digital accessibility in 4 dimensions—motivation, physical access, digital skills, and use—which tend to occur in succession. Digital skills can be divided into technology-based skills and digital literacy, where the ﬁrst skill focuses on the operation of digital devices and the latter focuses on creating, ﬁnding, processing, and communicating information. Digital literacy is more important than technology-based skills [50].

The emergent character of COVID-19 can be viewed as a motivation to search for information; however, because the pandemic started in territories distant from Spanish-speaking and Caribbean regions, the motivation was not immediate. Physical access to information was rapidly addressed by sources disseminated through Spanish-speaking countries, particularly Spain, Mexico, Colombia, Argentina, and Panama. However, the digital divide within Latin America and the Caribbean is more acute than in western countries, being worse for rural women [51], Indigenous communities [52], and people with disabilities, as early adopters use technologies to reinforce their privileged position [53]. Therefore, the numerous young generations eager to use new technologies and the development of decentralized access to digital content are fundamental for health communication in these regions.

Similarly, understanding the information and its context is more challenging for the abovementioned groups because of their lack of access to education, which translates into low levels of alphabetization. Thus, access to COVID-19–related information would be the ﬁrst step toward understanding this condition. The need for community-based support for accessing, interpreting, applying, and disseminating digital information must include medical institutions that directly manage and care for patients. Therefore, Twitter data from Spanish-speaking countries denoted the presence of hospitals, clinics, and medical associations addressing this issue. However, given the nature of COVID-19 as a global pandemic, it is necessary to improve the conditions of the public health infrastructure and staff in these regions.

### Contextualization of Health Information and Emergent Information Providers

Most research diffused in Spanish-speaking regions focused on WEIRD countries. This might be partly because Spain, a geographical location in terms of research cooperation with Europe [54], dominated the dissemination of COVID-19–related information in the Spanish language.

Park et al [54] investigated the global sharing of scientiﬁc papers using Altmetric data and determined that (1) most transcontinental sharing through Mendeley was from Europe, whereas most internal sharing was in the Asian and European clusters; (2) most transcontinental sharing through Twitter was also from Europe, whereas most internal sharing was in the African cluster; and (3) South American and other clusters accounted for the fewest or near fewest shares. Spain and Curacao were placed in the European cluster, which supports our finding that the sharing pattern of COVID-19–related, peer-reviewed sources in this country is atypical. Moreover, the Spanish-speaking countries considered in our study were included in the African, North American, and South American clusters, whereas the Caribbean countries were included in the North American, South American, and other clusters. If we combine both studies’ ﬁndings, we can note that language becomes more relevant when sharing emerging health information than previous scientiﬁc cooperation.

The status of English as an expert language should also be considered. English has been hegemonic in science and technology since the 18th century owing to British global commerce and the diffusion of the Protestant religion [55]. English-speaking countries were in optimal conditions in the 1950s, when the ﬁrst global computing developments occurred. Therefore, the new technology language by default was English, but this does not justify the fact that internet-based platforms often dismiss communication and information in other languages.

Several global conditions have been exacerbated by the COVID-19 pandemic, such as economic and social inequalities and indifference toward climate change. Meanwhile, gender violence and labor shortages are more prevalent in Latin America. This contributed to climate change, and the pandemic has affected immigration toward westernized regions, disrupting community networks and web-based connection patterns.

Thus, having a trusted health information source in a relevant context and language for Spanish-speaking patients is crucial. In cultures that are less hierarchical or distrust political authorities, a Facebook group managed by a local clinic, where the user or someone they know has been assisted, can influence the acceptance of emerging medical information.

Overall, we found few scientiﬁc papers related to COVID-19 nursing at the onset of the pandemic, suggesting a low perceived value of nursing care. Infection prevention and control in primary, community, and acute care settings were considered as key issues for nursing practices during the pandemic [56]. However, disinformation campaigns in the West and fear of the virus often translated to mistreatment of health care staff, and the Latin American context was no exception [57]. Thus, the value of nursing staff must be acknowledged not only through information but also through labor compensation, among other upgradation measures for the medical infrastructure in Spanish-speaking countries and regions that can improve patient care.

### Limitations and Future Studies

Regarding our data, altmetrics are an option to identify scientific studies but do not contain all available references. Regarding keywords, we did not use “sars-cov-2,” which is a more exact and scientific term than the “coronavirus” keyword used in this study. Most of the peer-reviewed resources examined in this study were not retracted at the time of analysis. However, COVID-19–related knowledge progresses rapidly worldwide; thus, the process of science self-correction might lead to increasing retractions. Our study only attempted to capture a speciﬁc moment of scientiﬁc information dissemination through social media in selected regions.

Given that Brazil was a relevant affiliation location for scientiﬁc papers circulating in Spanish-speaking regions, future studies should incorporate Portuguese and its related territories. Furthermore, peer-reviewed information circulation in Caribbean countries should be examined extensively, especially in the case of Curacao, as its COVID-19 information dissemination patterns do not correspond to other patterns in Spanish-speaking or Caribbean regions.

Considering the low internet penetration rate in some of our studied regions, the impact of offline social networks and health care networks on diffusing health information from web-based sources should not be discounted. Such hybrid networks should be investigated in detail in future studies. Given the internet beneﬁt gap between developed and developing communities [58] and the preparations required to facilitate the identiﬁcation and treatment of long COVID, the patterns of trustful health communication in Spanish-speaking and Caribbean communities must be explored at the microlevel and midlevel.

### Conclusions

We used Altmetric data about COVID-19–related information circulation in Spanish-speaking countries, with some mention of the nearby Caribbean regions. We considered the web-based sharing of scientific resources from multiple dimensions to provide their detailed characteristics and circulation patterns, considering language, geography, and accessibility factors in internetscapes. Such studies can enrich the methods for understanding scientific information in other underrepresented contexts, such as First Nations and communities with disabilities, and advance the management and analysis of web-based public data from non-white people to improve public health communication in their regions. We have presented our conclusions in Textbox 2.

**Summary of the conclusions.**

**Regarding the time dimension in Spanish-speaking and Caribbean regions**

- The highest information circulation peak for Spanish-speaking countries were from April 2020 to August 2020 and from December 2020 to April 2021, led by Spain.
- For Caribbean regions, the highest peak was from December 2019 to April 2020, led by Jamaica.

**Regarding Spanish-speaking territories at the beginning of the pandemic**

- Individuality dimension
  Scientific papers were the most mentioned resources.
  Most papers were related to medical and health sciences and available from their original publishers.
  Scientiﬁc expertise perceived as reliable was concentrated in a few peer-reviewed sources written in English.
- Place dimension
  The top scientiﬁc journals mentioned were from English-speaking westernized regions.
  Top scientiﬁc authorships were from China.
- Activity dimension
  The top mentioned scientiﬁc papers were related to breakthrough ﬁndings in the medical and health sciences area, written in highly technical language.
  Number of mentions did not always coincide with Altmetric scores.
- Relations dimension
  Top co-occurrences of authorship were self-loops in China.
  Top international collaborations were between China and the United States.
  South Korea and Singapore had high degree, whereas Singapore and Hong Kong had high betweenness.
  Argentina had high closeness and betweenness, and Spain had high closeness.
  Diverse actors, particularly from Panama, were relevant for the diffusion of peer-reviewed information.

## Appendix

Multimedia Appendix 1 Countries and regions selected for this study.

Multimedia Appendix 2 Publication (A) year and (B) month of peer-reviewed sources in Spanish-speaking countries.

Multimedia Appendix 3 Top knowledge areas of peer-reviewed resources.

Multimedia Appendix 4 Top sources on Twitter.

Multimedia Appendix 5 Journals that published mentioned (A) peer-reviewed resources and (B) countries of authorship.

## Abbreviations

WEIRD: westernized, educated, industrialized, rich, and democratic

## Data Availability

The data supporting the findings of this study are openly available over the internet [60].
