# Supplementary material for: Information Circulation Among Spanish-Speaking and Caribbean Communities Related to COVID-19: Social Media–Based Multidimensional Analysis
Source: J Med Internet Res. 2023 Aug 23;25:e42669. doi: 10.2196/42669 (PMC10448908; doi:10.2196/42669)
Supplement: Multimedia Appendix 1 [file jmir_v25i1e42669_app1.pdf]

Appendix 1. Selected countries and regions for this study.[a-c]

Name	Population	Spanish Status	Spanish Speakers	Percent	Category
Anguilla	15,174	Immigrant	-	-	C
Antigua and Barbuda	104,909	Immigrant	-	-	C
Argentina	44,688,864	Official	45,104,502	100.93	S
Bahamas	403,095	Immigrant	-	-	C
Belize	390,231	Spoken	195,597	50.12	C
Bolivia	11,215,674	Official	10,182,336	90.78	S
Bonaire	25,971	Spoken	125,534[b]	66.58	C
British Virgin Islands	32,206	Immigrant	-	-	C
Cayman Islands	63,129	Immigrant	-	-	C
Chile	18,197,209	Official	19,322,102	106.18	S
Colombia	49,464,683	Official	49,969,445	101.02	S
Costa Rica	4,999,384	Official	4,851,256	97.03	S
Cuba	11,492,046	Official	11,187,209	97.34	C
Curacao	162,547	Spoken	[c]	-	C
Dominican Republic	10,996,774	Official	10,302,220	93.68	S
Ecuador	16,863,425	Official	15,654,411	92.83	S
El Salvador	6,445,405	Official	6,745,456	104.65	S
Equatorial Guinea	1,402,984	Official	918,000	65.43	S
Guadeloupe	448,798	Immigrant	-	-	C
Guatemala	17,577,842	Official	15,599,542	88.74	S
Guyana	782,225	Spoken	-	-	C
Honduras	9,568,688	Official	9,039,287	94.46	S
Jamaica	2,906,339	Spoken	8,000	0.27	C
Martinique	385,320	Immigrant	-	-	C
Mexico	132,328,035	Official	125,875,402	95.12	S
Morocco	36,910,560	[d]	3,415,000	9.25	S
Nicaragua	6,351,157	Official	6,218,321	97.90	S
Panama	4,226,197	Official	3,504,439	82.92	S
Paraguay	6,896,908	Official	4,946,322	71.71	S
Peru	32,551,815	Official	29,541,922	90.75	S
Puerto Rico	3,654,978	Official	3,432,492	93.91	S
Saint Kitts and Nevis	56,345	Immigrant	-	-	C
Saint Martin	32,284	Immigrant	-	-	C
Sint Marteen	40,939	Immigrant	-	-	C
Spain	46,441,049	Official	46,158,388	99.39	S
Suriname	568,301	Spoken	-	-	C
Trinidad and Tobago	1,375,443	Spoken	65,886	4.79	C

Turks and Caicos Islands	36,461	Immigrant	-	-	C
Uruguay	3,469,551	Official	3,441,940	99.20	S
Venezuela	32,381,221	Official	32,214,158	99.48	S
Virgin Islands	104,909	Immigrant	16,788	16.00	C
Western Sahara	597,339	2nd Official	22,000	3.68	S

[a]Population from [34]; Spanish Status from [35, 36, 37]; Spanish Speakers from [38]

[b]With Curacao.

[c]See Bonaire.

[d]See Western Sahara.
