# Supplementary figures and images for: Information Circulation Among Spanish-Speaking and Caribbean Communities Related to COVID-19: Social Media–Based Multidimensional Analysis
Source: J Med Internet Res. 2023 Aug 23;25:e42669. doi: 10.2196/42669 (PMC10448908; doi:10.2196/42669)

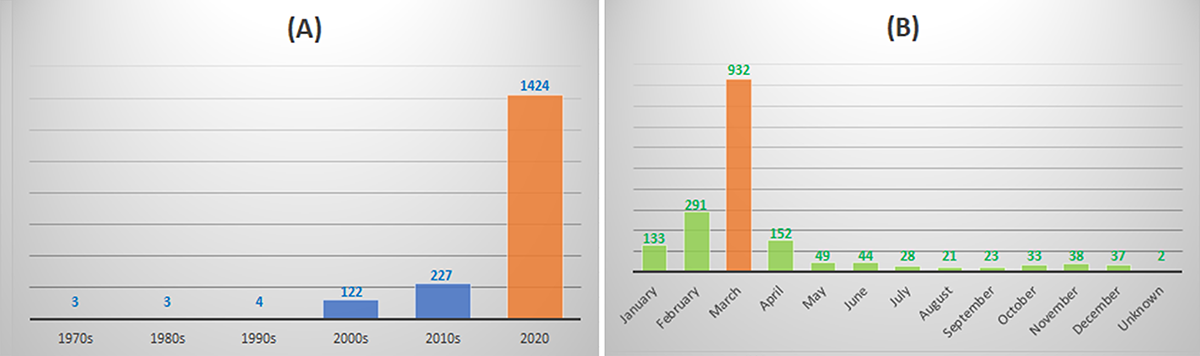

Supplement: Multimedia Appendix 2 [file jmir_v25i1e42669_app2.png]

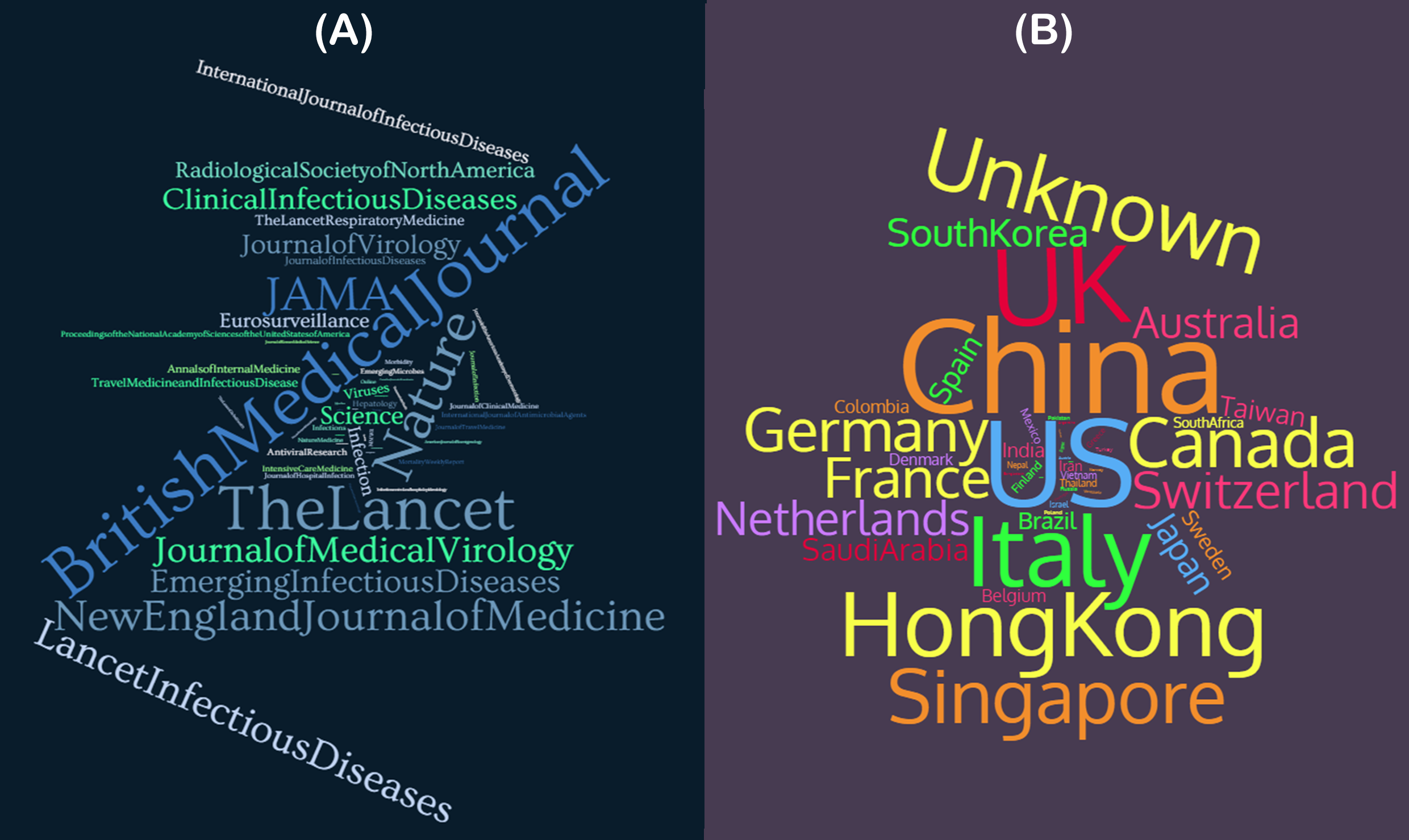

Supplement: Multimedia Appendix 5 [file jmir_v25i1e42669_app5.png]
