# Supplementary material for: Information Circulation Among Spanish-Speaking and Caribbean Communities Related to COVID-19: Social Media–Based Multidimensional Analysis
Source: J Med Internet Res. 2023 Aug 23;25:e42669. doi: 10.2196/42669 (PMC10448908; doi:10.2196/42669)
Supplement: Multimedia Appendix 3 [file jmir_v25i1e42669_app3.pdf]

Appendix 3. Top knowledge areas of peer-reviewed resources

Knowledge Area	No. of Resources	Percent
0011 Medical and Health Sciences	1,371	76.89
1103 Clinical Sciences	415	23.27
1108 Medical Microbiology	383	21.48
1117 Public Health and Health Service	380	21.31
0006 Biological Sciences	210	11.77
0605 Microbiology	76	4.26
1102 Cardiorespiratory Medicine and Haematology	69	3.86
0601 Biochemistry and Cell Biology	64	3.58
1107 Immunology	49	2.74
0604 Genetics	47	2.63
1199 Other Medical and Health Sciences	32	1.79
1114 Pediatrics and Reproductive Medicine	28	1.57
1112 Oncology and Carcinogenesis	18	1.00
1115 Pharmacology and Pharmaceutical Sciences	16	0.89
0017 Psychology and Cognitive Sciences	14	0.78
1109 Neurosciences	14	0.78
0002 Physical Sciences	11	0.61
0008 Information and Computing Sciences	11	0.61
0299 Other Physical Sciences	10	0.56
1110 Nursing	10	0.56
