# Supplementary material for: Information Circulation Among Spanish-Speaking and Caribbean Communities Related to COVID-19: Social Media–Based Multidimensional Analysis
Source: J Med Internet Res. 2023 Aug 23;25:e42669. doi: 10.2196/42669 (PMC10448908; doi:10.2196/42669)
Supplement: Multimedia Appendix 4 [file jmir_v25i1e42669_app4.pdf]

Appendix 4. Top sources on Twitter

Outlet Name	Tweets	Country	Category	Type
Sb Dirty South Socker	81	Panama	Media	Blog
El País	57	Spain	Media	Newspaper
Infobae	31	Argentina	Media	New Media
Sociedad Ecuatoriana de Alergia, Asma e Inmunología (SEAAI)	25	Ecuador	Association	Scientific Association
La Nueva España	23	Spain	Media	Newspaper
Agencia SINC	22	Spain	Media	News Agency
El Medico Interactivo	22	Spain	Media	News Media
El Espectador	20	Colombia	Media	Radio
La Voz Digital	20	Spain	Media	News Media
La Razon (ESP)	19	Spain	Media	Unknown
ABC desevilla	18	Spain	Media	Newspaper
ABC.es	18	Spain	Media	Newspaper
Colegio Médicos Postgraduados IMSS A.C.	15	Mexico	Education	Hospital
La Vanguardia	15	Spain	Media	Newspaper
Catalunya Vanguardista	14	Spain	Media	New Media
